# Time-Resolved Measurements of Turbulent Mixing in Shock-Driven Variable-Density Flows

**DOI:** 10.1038/s41598-019-56736-w

**Published:** 2019-12-30

**Authors:** John Carter, Gokul Pathikonda, Naibo Jiang, Josef J. Felver, Sukesh Roy, Devesh Ranjan

**Affiliations:** 1 George W. Woodruff School of Mechanical Engineering, Georgia Institute of Technology, Atlanta, GA 30332 USA; 2 Spectral Energies, LLC, Beavercreek, Ohio, 45430 USA

**Keywords:** Energy science and technology, High-energy astrophysics, Lasers, LEDs and light sources, Aerospace engineering, Mechanical engineering

## Abstract

Recent developments of burst-mode lasers and imaging systems have opened new realms of simultaneous diagnostics for velocity and density fields at a rate of 1 kHz–1 MHz. These enable the exploration of previously unimaginable shock-driven turbulent flow fields that are of significant importance to problems in high-energy density physics. The current work presents novel measurements using simultaneous measurements of velocity and scalar fields at 60 kHz to investigate Richtmyer-Meshkov instability (RMI) in a spatio-temporal approach. The evolution of scalar fields and the vorticity dynamics responsible for the same are shown, including the interaction of shock with the interface. This temporal information is used to validate two vorticity-deposition models commonly used for initiation of large scale simulations, and have been previously validated only via simulations or integral measures of circulation. Additionally, these measurements also enable tracking the evolution and mode merging of individual flow structures that were previously not possible owing to inherently random variations in the interface at the smallest scales. A temporal evolution of symmetric vortex merging and the induced mixing prevalent in these problems is presented, with implications for the vortex paradigms in accelerated inhomogenous flows.

## Introduction

The study of hydrodynamics relevant to high-energy-density physics (HEDP) frequently involves complexities and challenges in physics and engineering (reacting species, turbulence, mixing, plasma effects, electromagnetism, large spatial and temporal range of scales, extremely ‘fast’ physics, etc.)^1,2^. For example, the shock- and blast-induced instabilities, such as Richtmyer-Meshkov instabilities (RMI) and Rayleigh-Taylor Instabilities (RTI) play a dominant role in many applications, such as the mixing of fuel and air by incident shocks in a scramjet engine^3^, the ‘ignition problem’ of Inertial Confinement Fusion (ICF)^4,5^, blast-driven mixing of stellar media surrounding supernovae^6^ etc. Significant interest in these problems exists, with particular emphasis on effective models^5^ estimating the mixing effects of simulated hydrodynamics. Understanding and being able to model the shock-driven turbulent mixing of these phenomena is critical to further our understanding of HED physics. For example, a description of the fluid-interface growth in RMI is often provided by considering the vorticity generated by baroclinic torque at the interface due to the interaction of a pressure gradient (▽*P*, from shock) misaligned with a density gradient (▽*ρ*, from interface). This initial vorticity evolution was first modeled from linear stability theory^7^ and, for example, the Samtaney-Zabusky Model^8^ with good experimental agreement. Furthermore, Balakumar, *et al*.^9^ showed that the vortex configuration resulting from the initial shock advects material and neighboring vorticies (which strongly affects the extent of the mixing region) highlighting the importance of vorticity throughout the development of RMI. However, as secondary instabilities develop, the flow departs further from linear theory, and thus it is difficult to analytically describe the complex interactions which follow this regime into the transition and to turbulent mixing. Simulations have studied the evolution of the time-resolved vorticity field and found a significant change in vorticity due to additional production of the same by secondary instabilities^10^. There is currently no experimental work describing the temporal evolution of vorticity in RMI, much less in conjunction with density field evolution, to study the vortex interactions and induced mixing.

Early measurements of RMI in shock tubes used schlieren photography to primarily investigate the growth of the overall mixing layer^11^. These laid the foundation for understanding the linear and nonlinear growth regimes of the RMI. However, this is a volume-averaged method and planar techniques such as mie scattering, planar laser induced fluorescence (PLIF) and particle image velocimetry (PIV) have since become increasingly common providing more robust descriptions of the flow (such as mixed mass and turbulent spectra). Conventional research efforts on these fronts have used simultaneous measurements of velocity and density using large ensembles of uncorrelated (and independent) experimental runs with identical initial conditions. Recently a qualitative description of the morphological evolution of the interface and analysis of the growth rate of the mixing region have been provided in high-speed mie scattering and schlieren movies^12–14^. While these results do provide qualitative information about the mixing phenomena, their direct utility in providing benchmark validation data is limited. To this end, much pioneering work in the area of simultaneous PLIF and PIV has been performed^15–17^, albeit using large ensembles of snapshots from repeated experiments. In essence, simultaneous diagnostics are of interest to understanding the correlation between density and velocity fluctuations, and thus the turbulent mass-flux that is critical to closing RANS models such as the BHR model^18^ — a model extensively in use today for computations in variable-density turbulent mixing. Examples of such studies to enhance closure physics in variable density turbulence (albeit for low-speed classical RTI and KH instabilities) can be found in recent works^19,20^.

The challenges in experimentally studying and modeling such flows come primarily from the high spatial-resolution requirements inherently imposed by the typical mixing scales [~*O*(1−10 *μm*) in laboratory experiments], coupled with their extremely short temporal scales [~*O*(0.5−10 *μs*)]. These, together with the large scale flow evolution [~*O*(10 *cm*,10 *ms*)] lead to large desired dynamic ranges from the diagnostics. These requirements have conventionally limited the spatio-temporal detail with which these phenomena could be investigated. These illustrative ‘scale separations’ are for typical laboratory experiments, and the actual application could involve 2−10 orders larger separation than those seen in the laboratory. Recent advances in high-speed imaging and laser sources are paving the way for overcoming these limitations by employing non-intrusive optical measurements in complex reacting and non-reacting flow environments. For example, burst-mode lasers and gate-intensified CCD cameras are used to examine the velocity flow-field of supersonic flows by employing very high speed PIV at up to a *MHz*-repetition rate in compressible jet flows^21^, albeit at low resolutions and at considerable noise owing to the nature of the intensified imaging system. At conventional high-speed resolutions, Wernet^22^ and Beresh, *et al*.^23^ have made PIV measurements over hot and cold jets, supersonic jet in cross-flow and transonic flow over a cavity at acquistion rates ~*O*(10 *kHz*). Similarly, Wagner, *et al*.^24^ have implemented PIV in the wake of a cylinder in a shock-tube to study the harmonics of the transient wake growth. Besides PIV, the application of pulse-burst systems have been demonstrated for high-speed reactive- and passive-tracer PLIF (qualitative, Michael, *et al*.^25^, for eg.), thermometry^26^, etc. Additionally, akin to the current work, Miller, *et al*.^27^ have demonstrated the ability for simultaneous velocity and qualitative-PLIF measurements at 10 *kHz* using pulse burst systems. Numerous other application-specific implementations of high-speed diagnostics have been presented in the thorough review of Thurow, *et al*.^28^ The current work extends this spatial and temporal dynamic range [≈*O*(100 *μm*−100 *mm*) and ≈*O*(10 *μs*−10 *ms*)] simultaneously in velocity and concentration/density fields to enable studies of shock-driven mixing-related physics that were previously not possible due to insufficient dynamic range.

We leverage and demonstrate the aforementioned developments in high speed instrumentation to perform simultaneous velocity - species concentration measurements to study the evolution of Richtmyer-Meshkov Instability (RMI) in an inclined shock tube and subsequent turbulent mixing induced by the same. For this, we configure a dual-leg pulse-burst laser and two high-speed Photron SA-Z Fastcam cameras operating in a synchronized manner. In burst-mode lasers, a low-energy continuous wave laser is sliced into a sequence of pulses which are then amplified to produce pulse doublets in close succession for a limited amount of time, which typically lasts 1–100 *ms* depending on the laser architecture^28^. A high-speed planar PIV and acetone-PLIF system is designed to capture the physics of RMI in a time-resolved sense for the first time. Specifically, we aim to demonstrate the ability to measure the turbulent structure in sufficient detail to make comments on scalar mixing in space and time, and to indicate ways in which such instrumentation can be leveraged for a better understanding of mixing phenomena.

## Design of Experiments

### Richtmyer-Meshkov Instability (RMI) and flow facility

Richtmyer-Meshkov instability (RMI) typically ensues when a shock wave propagates from one medium into a medium of different thermodynamic properties (density & ratio of specific heats). The propagation, when coupled with small misalignments between the interface and shock-front, results in a ‘baroclinic deposition’ of vorticity on the interface. This is governed by the vorticity equation, which, for fluids initially at rest, is given by $D\omega /Dt=\frac{1}{{\rho }^{2}}\nabla \rho \times \nabla P$. It can be seen that the amount of vorticity deposition, and thus the Reynolds number of the instability, increases with increase in density gradients (‘Atwood number’), pressure gradients (shock strength or Mach number, *M*) and the mismatch angle between the two gradients (as sin*α*). The subsequent evolution of the instability involves vortex stretching and transport of this impulsively deposited vorticity (and consequently the species) via the classical vorticity transport and instability mechanisms. For ‘low’ shock strengths (typically $1\lesssim M\lesssim 3$) and simple interfaces (typically planar), complexities such as secondary interactions with reflected and refracted shocks, vorticity production at vortex cores, compressibility effects in instability evolution, etc. can be ignored. The current experiments are designed in this parameter space where a planar shock and a planar interface that are misaligned at a constant angle is studied. This frequently forms a canonical configuration for numerical, experimental and theoretical analyses to understand fundamental mechanisms such as the vortex-roll up, initial condition memory, linear and non-linear growth mechanisms, etc. While a brief description on the experimental configuration to study this is provided below, more details on the physical facility can be found in Mohaghar, *et al*.^29^. Likewise, a more thorough review of RMI and its relevance to the many HEDP phenomena is presented in the recent summary of Zhou^4^.

**Figure 1:**
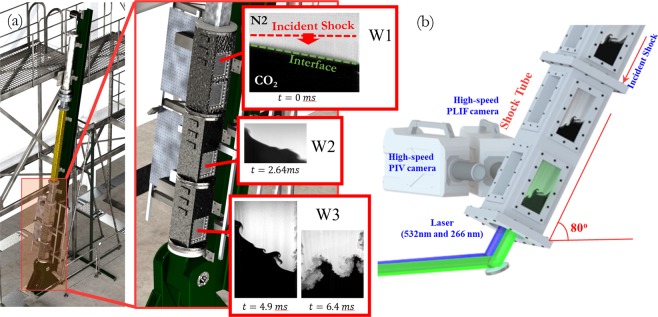
Schematic of the inclined shock-tube on which the current experiments were performed. Also shown are the measurement windows (W 1–3), illustrative interface states and a schematic of optical setup for the diagnostics.

The current experiments are designed to make simultaneous planar measurements of velocity and concentration (and hence density) at high temporal resolutions, to investigate a shock-driven instability growth between two gasses (*N*_2_ and *CO*_2_) and the subsequent turbulence-induced scalar mixing. Experiments were performed at the Shock Tube and Advanced Mixing Laboratory (STAM Lab) at Georgia Institute of Technology, with a thorough description of the facility available in our previous works^30,31^. The entire tube has the ability to incline with the ground, which enables an easy control of the misalignment between the shock front (oriented along shock-tube cross section) and a stably stratified horizontal interface (oriented perpendicular to the gravity). A stably stratified interface is maintained at 1.7*m* from the bottom end of the shock-tube by filling a heavy gas (*CO*_2_ here, *ρ*_2_ = 1.84 *kg*/*m*^3^) from the bottom, and a light gas (*N*_2_ here seeded with acetone, *ρ*_1_ = 1.16 *kg*/*m*^3^) from the top, giving an effective initial Atwood number $(At=\frac{{\rho }_{2}-{\rho }_{1}}{{\rho }_{2}+{\rho }_{1}})$ of 0.22. The shock-tube test section with the interface and incident shock configuration is shown in Fig. 1. The test section allows optical access at each module through staggered windows (*W*1−3 as shown) to record the development of the perturbed interface advecting from initial to the latest experimental times. A polycarbonate diaphragm at the top of the shock-tube sandwiched between the driver and driven sections is ruptured by pressurizing the driver section to 110*PSI* using *N*_2_ gas. The rupture causes a planar shock front at Mach (*M*) = 1.55 that impinges on the inclined interface between the two gases (at window location *W*1), and deposits baroclinic vorticity on the interface. The Mach number is measured experimentally using two high-dynamic pressure transducers mounted downstream of the X-plate, and the gas properties are calculated using 1-dimensional gas dynamics equations^32^. The shocked interface evolves and convects behind the incident shock through windows *W*2 and *W*3, before the shock-wave rebounds on the bottom wall and impinges on the interface a second time. This second interaction (termed ‘re-shock’) at viewing location *W*3 deposits additional vorticity on the convecting interface, halts the convection, and transitions the instability to turbulence to initiate rapid mixing^29,33^. Here, simultaneous PIV and PLIF of acetone at 60 kHz are employed to investigate this interface after first shock (at two physical windows, W1 and W2), and the evolution and mixing of the interface after reshock (at bottom window, W3). Figure 1a shows the test section module, together with an example interface (from PLIF imaging) as it evolves downstream.

### Laser configuration and architecture

**Figure 2:**
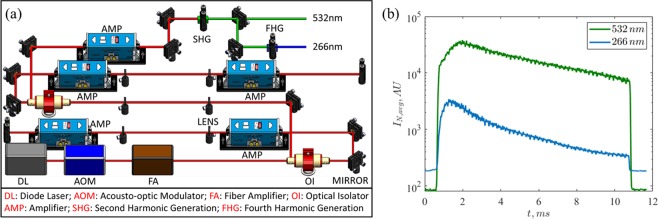
(**a**) Architecture of the burst-mode laser system, with the various components, and (**b**) laser power changes in 532 *nm* and 266 *nm* outputs as measured by mean intensity of PIV and PLIF images.

A specially configured burst-mode laser is used as the light source for both PIV (at 532 *nm*) and PLIF (at 266 *nm*). The general architecture of the burst-mode laser has been described in detail in other works^34–36^, so only a brief overview will be given here noting specific modifications for simultaneous diagnostics. The specific configuration used for the current experiments is shown in Fig. 2a. A flexible master oscillator is used that can produce tunable pulse widths by slicing the output from a 30 *mW*, 1064 *nm* continuous-wave, narrow-band diode laser. A 10 *GHz*-bandwidth, Acousto-Optic Modulator (AOM) with an extinction ratio > 50*dB* is used to perform the pulse splicing. The AOM is modulated by a pulse generator which can be controlled using the software. The sliced 1064-nm pulse train is amplified in stages, and final laser output can reach laser energies ≈1*J*/*pulse* at 1064 *nm* at a repetition rate of 10 *kHz* and a burst duration of 10 *ms*. At 100 *kHz* repetition rate, the average laser pulse energy reduces to ~100 *mJ*/*pulse*. The 1064 *nm*-laser is frequency doubled using a second harmonic crystal to produce 532 *nm* light for PIV, and then doubled again to produce the fourth harmonic 266 *nm* light for PLIF. For the current experimental conditions, a short burst of 532 *nm* and 266 *nm* pulses at 60 *kHz* provided for about 10 *ms* is sufficient to study the development of the interface, although the laser could be configured to operate at up to 1*MHz*. The peak laser pulse energies respectively were 60 *mJ* and 25 *mJ*, respectively.

The laser beam from the output is directed into the the shock-tube via a series of dual-wavelength mirrors and beam-shaping optics to provide a sheet with a beam waist of ≈500 *μm*. The light sheet enters the shock tube from the bottom end plate through a sapphire glass window, and slices the interface at the measurement locations. The focal-length and location of the cylindrical lens that generates the sheet were adjusted depending on the measurement location (windows, W1, W2 or W3) so as to optimize the amount of laser energy available for PIV and PLIF in the field of view.

### Imaging and diagnostics

Two Phantom SA-Z CMOS cameras were mounted next to each other on a rail that enables moving the cameras along the shock tube depending on the window to be imaged. While the PIV camera was viewing perpendicular to the light sheet, the PLIF camera was mounted with a tilt (≈15°) and a scheimpflug mount viewing the same field of view as that of the PIV camera. Images of a calibration target were taken with both cameras at each imaging window to enable spatial calibrations and registration between the two fields. The CMOS cameras are capable of imaging at up to 20 *kHz* at a full resolution of 1 megapixel. To temporally resolve the flow in current experiments, the cameras were run at a reduced resolution of 0.3*MP* but sampled at 60 *kHz*. Both cameras were fitted with Nikon 50 *mm* lens, with a maximum aperture of *f*/1.6. The depth of focus of the imaging optics was around 5 *mm* (at 266 *nm*) which is much larger than the laser sheet thickness.

To enable simultaneous measurements of velocity and density, both the gasses in the shock tube were seeded with 0.3−1 *μm* TiO2 particles for PIV, while the light gas (*N*_2_) was additionally seeded with Acetone vapors. While the particles scatter the incident 532 *nm* light, the acetone vapors absorb the 266 *nm* light and fluoresce in the visible UV range (scattered 266 *nm* light from particles is weak and is blocked by the acrylic windows that only allows visible light). Additionally, to enable simultaneous imaging of the mie-scattered 532 *nm* light from the seeded particles and fluoresced light from the seeded acetone, the PIV camera was fitted with an Omega model 532BP10 532 *nm* band-pass filter (OD ≥ 3) transmitting only scattered light, and the PLIF camera images through an Edmund Optics TECHSPEC 532 *nm* notch-filter (OD ≥ 4) that blocks the scattered light.

The PIV particle images were processed using https://www.lavision.de/en/products/davis-softwareLaVisionDaVis8.4 software, using recursive grid refinement to a smallest window of 24 × 24*px* (50% overlap). This, given the magnification of the imaging, gives a spatial resolution of 4 *mm*/*vec*. A 3 × 3 × 3 spatio-temporal median filter was used for outlier detection, vector replacement and Gaussian smoothing (to remove high frequency noise) in the PIV vector fields. The PLIF images presented in the current work are qualitative in nature and have a spatial resolution of 250 *μm*. The mean decay in laser intensity is corrected using a median normalization given by,

1 $${I}_{c}=\frac{I-{I}_{\min }}{{I}_{med}-{I}_{\min }}$$

2 $${I}_{N}=\frac{{I}_{c}}{{I}_{c,avg}}$$

where *I*_*N*_ and *I*_*c*_ are normalized and median-corrected images, *I* is the raw image, *I*_*min*_ and *I*_*med*_ are the minimum and median intensities of the image, and *I*_*c*,*avg*_ is the average intensity of *I*_*c*_. This median based normalization results in accurate corrections for laser intensity, and is immune to outliers in individual images.

### Experimental considerations

An important consideration for the current experimental setup was balancing the energies between the two laser beams for optimization of signal-to-noise ratios in both PIV and PLIF applications. There were two constraints that were considered in designing the current system. Firstly, since both the laser beams (532 *nm* and 266 *nm*) originate from the same fundamental laser (at 1064 *nm*), their energies are coupled to each other. Owing to inefficiencies typically present in doubling the frequency, the fourth harmonic beam (266 *nm*) inherently has lower energy than the second harmonic counterpart (532 *nm*). Further, the peak power that the laser produces at 266 *nm* at 60 *kHz* (≈25 *mJ*/*pulse*) is lower than those typically available from low speed, high energy (≈100 *mJ*/*pulse* at 1*Hz*) Nd:YAG lasers. Secondly, the energies of the beams reach a peak shortly after the beginning of the emission, and slowly decay at later time. These effects can be seen in Fig. 2b, which shows the average intensities of PIV and PLIF raw images with time. This implies that the signal to noise ratio, especially in PLIF images, is lower at the end of acquisition than at the beginning.

To work around these constraints, PLIF images are first optimized by maximizing the energy of the fourth harmonic and by increasing the aperture of the PLIF imaging system. This however leads to over exposure of the PIV images even at lowest aperture (high *f*/#), thus drastically increasing the peak-locking of the PIV vector fields^37^. This was mitigated by using a series of neutral density filters in front of the camera to reduce the intensity of the particle images to within the dynamic range of the camera. This still leaves the late time PLIF images (>8 *ms*) highly qualitative owing to the comparable strengths of PLIF signal and CMOS sensor noise. This effect is significant even at earlier times at regions where there is strong absorption of the excitation light as it travels through *N*_2_. This impact could be mitigated by using a higher power burst-mode laser that is currently under construction.

## Results and Discussion

### Comparison to high-resolution experiments

**Figure 3:**
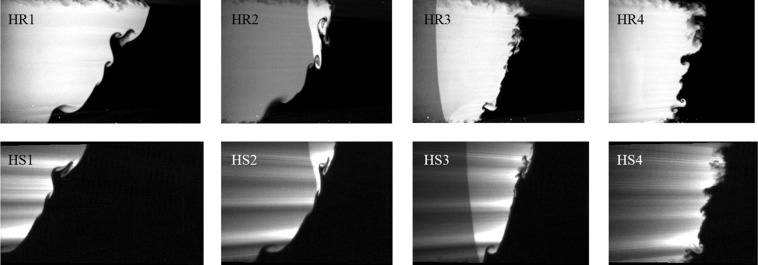
Qualitative comparison of interface images captured employing low-speed, high-resolution (HR, from Mohaghar, *et al*.^33^) PLIF and current high-speed moderate resolution (HS) PLIF are shown at four times (1−4, 5.0,5.2,5.3,5.5 *ms* after incident shock). The passage of reshock from right to left can be seen in images 2 and 3.

The processed high-speed PLIF images show excellent agreement with previous high-resolution observations^33^ on the same physical facility, especially at more deterministic large scales. This can be seen in Fig. 3 where the the PLIF mixture fraction fields from high-resolution experiments at W3 are compared to the images of the corresponding high-speed case at various evolution times. Supplementary video S1 shows the full evolution of the interface at the three different windows *W*1−*W*3 from the current experiments (note that the three views in *W*1−*W*3 are from different experimental runs). Note that while the previous data was acquired from separate experiments (one field per experiment-time), the current data shown is acquired from a single continuous progression of the interface. This enables tracking the evolution and mode merging of individual flow structures that were previously not possible owing to random variations in the interface growth at the smallest scales. Further, the current experimental data also enables the study of interface compression due to reshock (times 2–3 in Fig. 3), phase inversion of mean interface evolution, and the continuous evolution of the small scale features (time 4 in Fig. 3). The complete progression as a video can be seen in the Supplementary Video S1, which shows details of the evolution of small turbulent vortical scales and the turbulent mixing of the two gasses by the instability mechanisms. Also evident at late times in the supplementary video S1 is the aforementioned reduction in laser energy at late times, and the associated decrease in signal-to-noise ratio in the PLIF results.

### Velocity and vorticity dynamics

**Figure 4:**
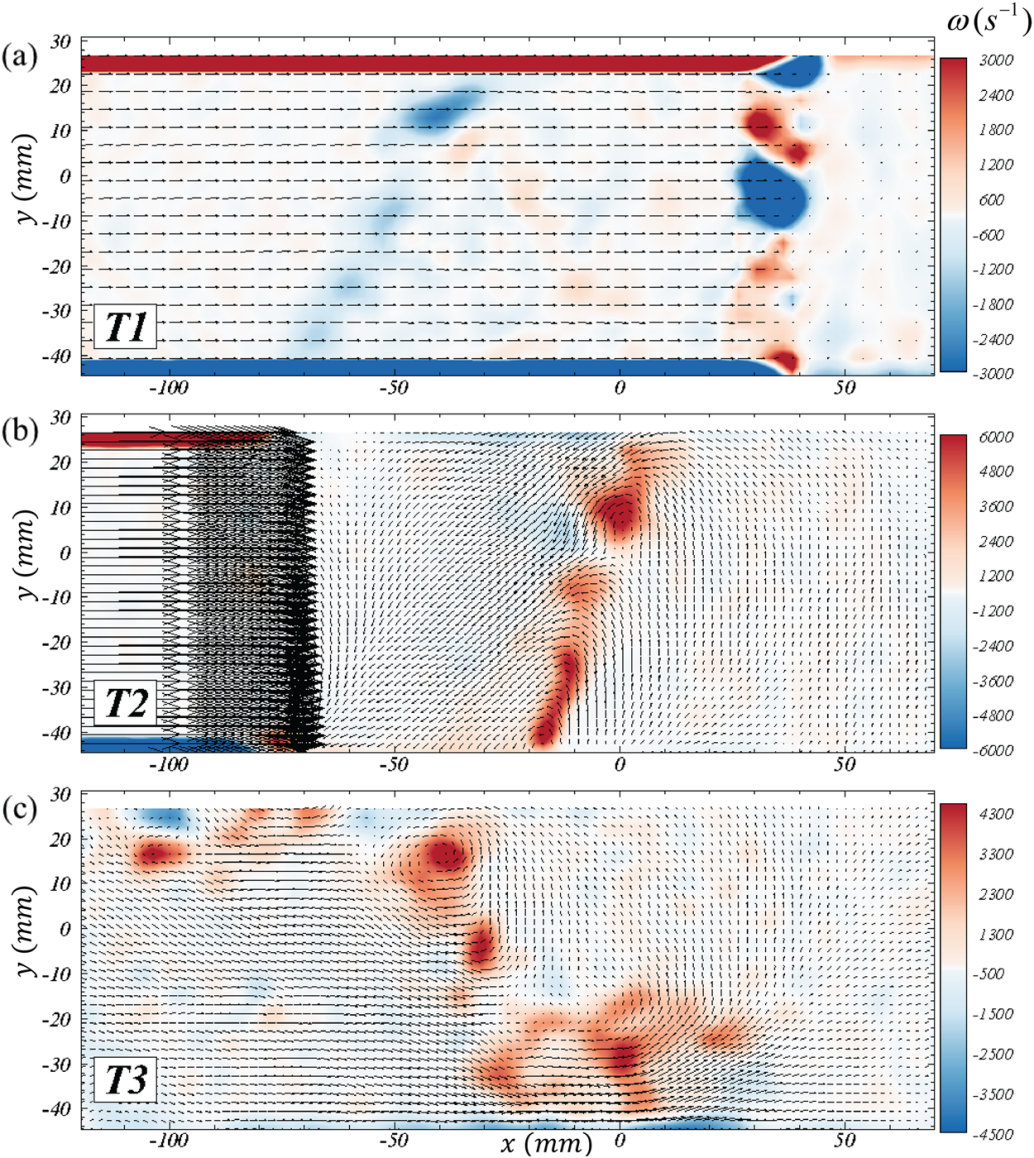
Evolution of vorticity (*ω* in *s*^−1^) at three different times corresponding to (**a**) late-time pre-reshock (*T*1 = 4.9 *ms*), (**b**) early time post-reshock (*T*2 = 5.3 *ms*) and (**c**) late time post-reshock (*T*3 = 7.5 *ms*). Velocity vectors are scaled to 1% of magnitude in (**a**) and to 10% in (**b**) and (**c**). See Supplementary video S2 for the full evolution in W3.

Richtmyer-Meshkov instability is primarily a vorticity-driven phenomenon, and the current experiments provide valuable information on the evolution of the same. Figure 4 shows the velocity fields overlaid on the vorticity contours at three important times of the evolution - late time interface after incident shock (*T*1 = 4.9 *ms* from incident shock), early time interface after reshock (*T*2 = 5.3 *ms*), and late time after reshock (*T*3 = 7.5 *ms*). Contour levels of magnitude <8% of peak are not shown to avoid small-scale noise in gradients and for clarity. The wholly negative orientation of deposited vorticity at T1 (from the initial orientation between interface and incident shock) is accurately captured. Also captured at T1 is the noise in vorticity from spurious vectors at the reshock front ($30\,mm\lesssim x\lesssim 40\,mm$, due to refraction of particle signal) and near the walls (*y* > 20 and *y* < −40, due to large shear). As the vortex sheet is stretched, interface at T1 develops the vortex reorganization into pockets of concentrated clockwise vorticity. This also manifests as strong perturbations in shape of the interface as the reshock deposits additional vorticity on the same at T2. The phase inversion and strengthening of these vortices (owing to the additional deposition and greater mismatch) is evident immediately after reshock. The subsequent modal evolution, vortex merging and breakdown leading to a rich turbulent mixing environment is evident at T3. Supplementary video S2 captures this detailed progression, and is the first measurement of the high-speed RMI evolution. The final interface velocity field shows strong intermixing and mean scalar transfer of each gas into the other.

**Figure 5:**
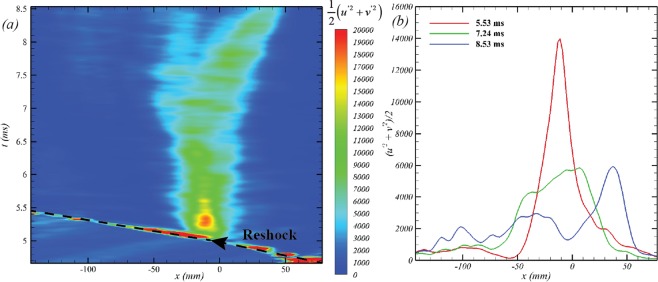
(**a**) Temporal evolution of two-dimensional, spanwise averaged TKE estimate and (**b**) the streamwise redistributions of the peak energy at different evolution times after reshock.

The turbulent kinetic energy on the interface ultimately responsible for turbulence is deposited by shock at very small length scales [~*O*(diffusion thickness) ≈ *O*(*mm*)]. The vortex stretching and inviscid instability mechanisms redistribute this turbulent kinetic energy spatially and spectrally leading to a rich range of velocity (and consequently scalar) scales. The two-dimensional estimate to the turbulent kinetic energy (*TKE*_2*D*_) can be obtained via the in plane velocity fluctuations as the flow is statistically homogenous in the *z*−direction. Assuming reflective symmetry of the initial conditions and spatial homogeneity in the spanwise direction, Fig. 5 shows this measure from fluctuating velocities computed via spanwise averaging. Immediately after the interaction with the reshock, a localized peak in turbulent fluctuations is observed. This peak TKE grows spatially across the interface as the classical instability mechanisms redistribute the same. Further, the total *TKE*_2*D*_ in the FOV remains relatively constant owing to the negligible dissipation relative to redistribution mechanisms (this value starts to show a decay-trend at late times for *t* > 8 *ms*). These demonstrate the strong inverse cascade of energy expected at early times of the instability growth without a significant dissipation.

Equivalently, the primary momentum for turbulence and mixing comes form the deposited baroclinic vorticity from the shock-interface interactions during incident shock and the reshock. Many studies studying the growth, transition and mixing of the RMI using computational methods approach the problem as two separate processes – (1) the shock-interface interaction (responsible for initial vorticity deposition), (2) and the subsequent evolution of the same (responsible for mixing). This decouples the compressibility effects involved in RMI initiation from the subsequent hydrodynamics, which can then be analyzed using divergence-free field analyses (Biot-Savart time-integration^38^, for eg.). The mechanics of the shock-induced vorticity-deposition has been done in many previous studies and depends on the problem geometry (interfaces vs bubbles^39,40^), shock-propagation direction (light-heavy vs heavy-light^40–42^), strength of secondary waves (reflected and refracted shocks and rarefaction waves^41^), etc. The availability of high-speed measurements of PIV and PLIF enable us to study the vorticity-deposition models and provide direct experimental information to evaluate the same. The current experiment provides two cases for the evaluation of such models - interaction of the incident shock with the inclined interface and (a short time later) the interaction of the reshock with the interface that has now slightly evolved to a more complex shape. In the following analysis, we use normal symbols (*ρ*_1,2_, *c*, etc.) to refer to unshocked properties, *prime* (′) annotations for once-shocked properties (after passage of incident shock), and *double-prime* (″) for twice-shocked properties (after passage of reshock). Additionally, the subscripts 1 and 2 correspond to light (*N*_2_) and heavy (*CO*_2_) gasses respectively. These properties are obtained via 1-dimensional gas dynamics analysis which are detailed in the works of Mohaghar^32^.

**Figure 6:**
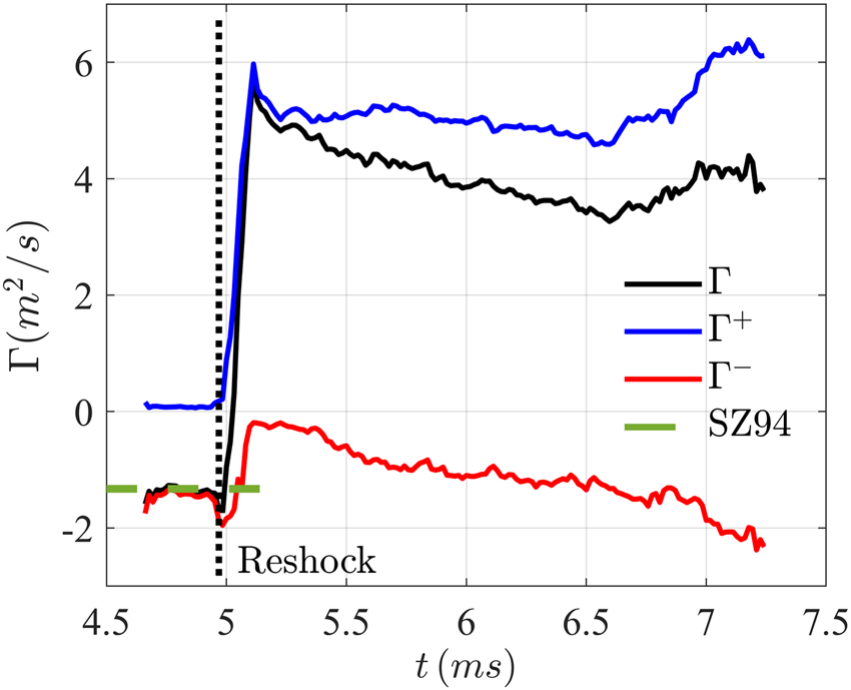
Evolution of the circulation of the interface by net vorticity, positive-vorticity and negative vorticity.

Figure 6 shows the evolution of the net circulation on the interface seen in W3. The interface as viewed in this location is already shocked by the incident shock as it enters the field of view, and thus starts with a non-zero circulation (net-negative vorticity here). Assuming no dissipation of deposited vorticity, the measured value of this circulation is compared with the shock-polar model of Samtaney and Zabusky^40^ (hereafter referred to as SZ94) which estimates the deposited circulation (*σ*) per unit interface length as

3 $$\frac{\sigma }{{c}_{1}/\sqrt{\gamma }}=(1-{\eta }^{-\frac{1}{2}})(\sin \,\alpha )(1+{M}_{i}^{-1}+2{M}_{i}^{-2})({M}_{i}-1)({\gamma }^{\frac{1}{2}}/\gamma +1)$$

here, the *c*_1_ is the speed of sound in un-shocked nitrogen, *η* = *ρ*_2_/*ρ*_1_ > 1, *γ* is the mean ratio of specific heats, *α* is the misalignment between the interface and the shock (10° here), and *M*_*i*_ is the *M*ach number of the incident shock. This comparison shown in Fig. 5 at *t* < 5.0 *ms* shows a remarkable agreement with the estimated circulation on the interface. Also shown in Fig. 6 are the net positive (Λ^+^) and negative (Λ^−^) circulation evolutions with time. Bulk of the circulation comes from the reshock, which quickly reorganizes owing to the unstable nature of the interface. This reorganization happens with negligible dissipation (shown by near-constant evolution of Λ^+^), though the net circulation reduces in time. This linear decay in net vorticity is entirely consistent for small-inclination interfaces, as was previously noted in the computations of McFarland, *et al*.^43^, and arises due to a flux of negative vorticity from the wall-vortex (clockwise oriented) that detaches from the wall towards the center of the shock-tube^42^. The complex interaction of the interface deposited vorticity and the wall-vortex at late times (*t* ≈ 6.5 *ms*) results in a net increase in both clockwise and counter-clockwise vorticity, as can be seen in supplemental video S2.

More importantly, we can also utilize the late-time pre-reshock interface configuration (at time *t*_*r*_^−^, Fig. 2a) measured using PLIF images and the velocity fields after reshock (*t*_*r*_^+^, Fig. 2b) to study the net vorticity-deposition by the reshock. For this analysis, the shock propagation configuration is ‘slow-fast’ in nature (heavy-to-light gas), deposition is assumed to be *regular*, and occurring over an interface of ‘small’ perturbations (*A*/*λ* ≤ 0.1, where *A* is the perturbation amplitude, and *λ* is perturbation wavelength^42^). We evaluate two models commonly used for vorticity initiation - (a) one based on shock-polar analysis and asymptotic treatment of shock refraction effects (Samtaney, *et al*.^42^, hereafter referred to as SRZ98), and (b) model based on integration of baroclinic torque, a first order approximation of impulsive acceleration and one-dimensional gas dynamics assumptions (Weber, *et al*.^38^, hereafter referred to as WCB2013). The reader is referred to the original works^38,42^ for more details of the models. We invoke the assumptions of ‘regular’ shock-refraction (ignoring the reflected and refracted shock and rarefaction effects), small perturbation amplitudes, impulse density gradients in the shock-propagation-direction at the interface, and a single-valued nature of the interface profile (at locations where this is strictly not obeyed, we consider the interface location representing the strongest density gradient in shock-propagation direction). Additionally, the interface is assumed to have infinitesimal width, and all the vorticity is assumed to be concentrated as an impulse function around it. Under these assumptions, SRZ98 model can be summarized for the current case as

4 $$\frac{\sigma (s)}{{c}_{2^{\prime} }/{\gamma }^{1/2}}=\frac{2}{{\gamma }^{1/2}}(1-\frac{1}{\sqrt{\eta ^{\prime} }})\xi ({M}_{r})\sin [\alpha ^{\prime} (s)]$$

where *σ*(*s*) is the deposited circulation per unit length along the interface coordiante (*s*), *c*_2′_ is the sound speed in heavy gas, *η*′ = *ρ*_1′_/*ρ*_2′_ < 1, *α*′(*s*) is the mi*s*match angle between the local tangent of interface and the reshock, *M*_*r*_ is the *M*ach number of reshock, and

5 $$\xi ({M}_{r})=\frac{4\gamma }{\gamma +1}({M}_{r}-1).$$

**Figure 7:**
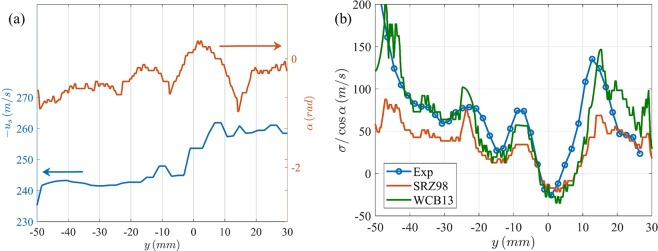
(**a**) The interface jump velocity and the misalignment of interface with reshock along the shock tube width, and (**b**) comparison of deposited circulation per unit shock-tube-width with the models of SRZ98 and WCB13.

Similarly, the estimated reshock-deposited-vorticity from the WCB13 can be rewritten for the current assumptions as

6 $$\omega (s)=\frac{{u}_{s}(s)({\rho }_{2^{\prime} }-{\rho }_{1^{\prime} })(-\,\tan \,\alpha )}{\frac{1}{2}({\rho }_{1\text{'}}+{\rho }_{2^{\prime} })}$$

here *u*_*s*_ is the jump in interface velocity from before to after reshock (note that there is a ratio of impulse function to its heavyside function that evaluates to unity at the interface and has dimensions of 1/length scale). The orientation of the interface immediately before its interaction with reshock forms the initial condition for the models. The 2-dimensional interface shape *s*(*y*) before reshock is extracted from the PLIF images at *t* = *t*_*r*_^−^ to compute the local angle [*α*(*s*)] with respect to reshock (reshock is assumed to be vertical). The jump velocity, *u*_*s*_(*s*) = *u*_*s*_^+^−*u*_*s*_^−^ of the interface from reshock is also measured from the pre-reshock and post-reshock scalar and velocity fields (*u*_*s*_^−^ and *u*_*s*_^+^ respectively). The variation of these two quantities along the shock-tube width (and along the interface, owing to its single-valued nature) is shown in Fig. 7a.

Finally, the distribution of net circulation per unit interface length is computed from the measured vorticity field, *ω*(*x*, *y*, *t*) as

7 $$\sigma (s)=\,\cos \,\alpha {\int }_{s(y)-\Delta /2}^{s(y)+\Delta /2}\,(\omega (x,y,{t}_{r}^{+})-\omega (x,y,{t}_{r}^{-}))dx$$

where Δ is an arbitrary neighborhood around the interface to fully encompass the measured/diffuse vorticity field.

Figure 7b shows the comparison between the predicted circulation deposition per unit shock tube width, (*σ*/cos*α*), by the two aforementioned models together with the experimentally measured counterparts. The figure shows a remarkable agreement between the two models and the experiments, justifying both the approaches for these conditions. It serves to note that the SRZ98 model as is presented here utilizes only the measured shape [*s*(*y*)], and implicitly estimates the jump velocity (*u*_*s*_) based on 1-dimensional gas dynamics relations. The WCB13 model, however, directly utilizes the measured jump velocity profile across the interface together with the interface orientation relative to the shock. This latter approach offsets some of the assumptions made in the current work, which lead to the discrepancies. For example, the assumption of single interface interaction (that the interface is a single valued function) is strictly not valid everywhere (10 *mm* < *y* < 20 *mm*, for eg.), and the ability to specify a jump-velocity profile in WCB13 from experiments improves the estimated vorticity deposition in the corresponding region. This deposited circulation serves as the initial condition for subsequent instability growth, transition and mixing dynamics. While many computational studies have investigated the ability of these models to represent the deposited baroclinic vorticity^38,42,44^, for eg.], the current work experimentally measures the interface orientation and the vorticity before and after reshock in a time-resolved manner to provide a direct validation of slow-fast vorticity deposition.

### Small-scale mixing dynamics from simultaneous measurements

**Figure 8:**
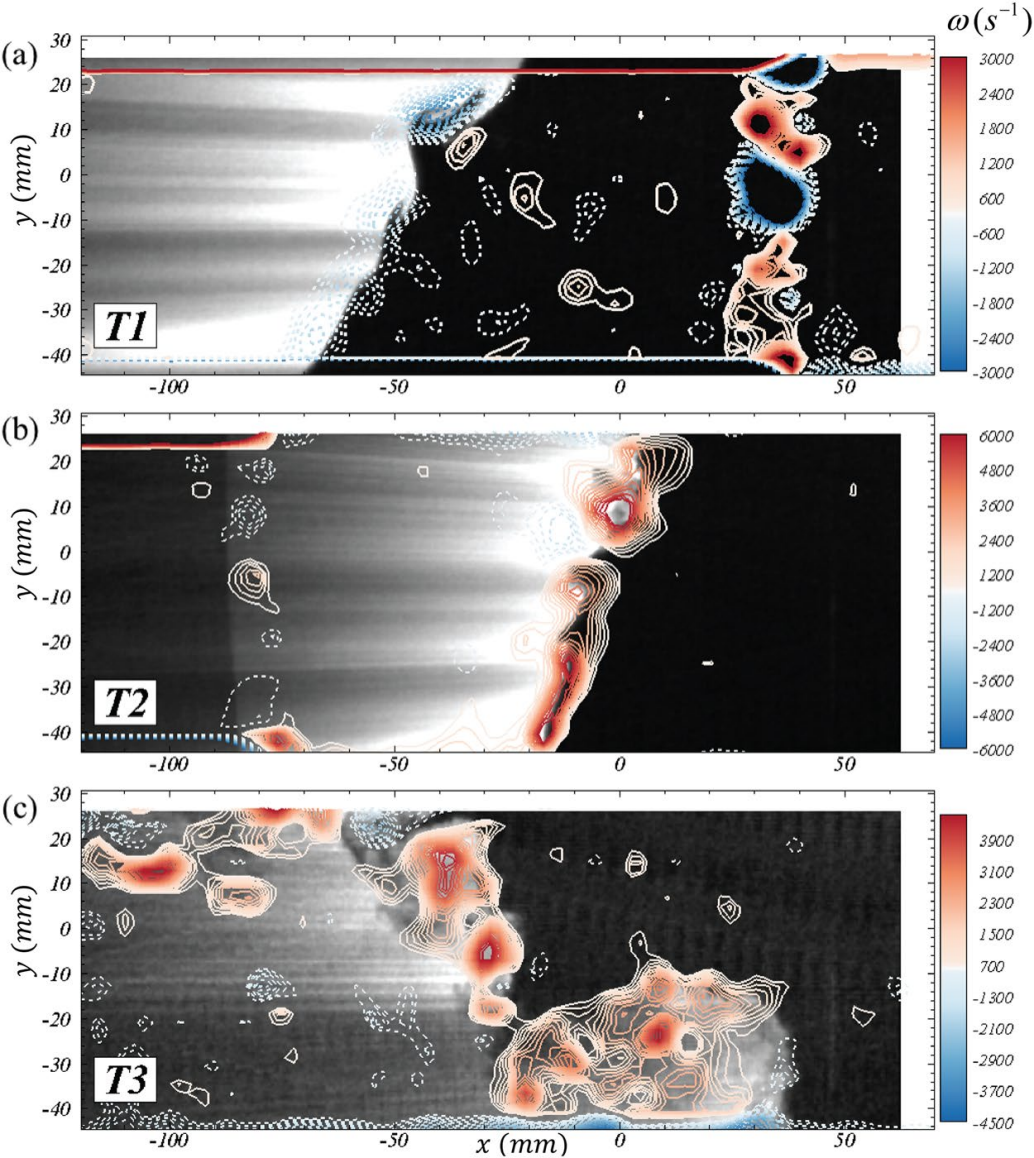
The vorticity-dominanted-mixing characteristics of RMI shown via contours of vorticity (*ω*) and corrected PLIF intensities (*I*_*N*_). (**a**–**c**) correspond to same times *T1–T3* described in Fig. 4. The mixing activity of the gasses is concentrated as pockets of strong vorticity (in *s*^−1^). See Supplementary video S3 for the full evolution in W3.

The goal of the current simultaneous diagnostics is to visualize the effect of turbulent velocity scales and mixing simultaneously. Figure 8 shows the mixing induced (via PLIF images) by the vortical activity represented by the contours of vorticity. The Supplementary video S3 shows the continuous time version of the figure, elucidating the coupling between the mixing and vortical activity in an RMI. Pre-reshock, it can be seen that the deposited vorticity organizes itself into pockets of concentrated vortical structures, as the associated perturbations grow. The current work shows a clear lack of mixing transition^45^ at this time, with very little mixing activity between the two gases. This late time interface before reshock at T1 forms the initial condition for the subsequent vorticity deposition by reflected shock at T2. A phase reversal is noticed as the opposite signed (positive) vorticity deposition occurs, which leads to strong vortical structures. Most of the intermediate-time turbulent mixing post-reshock is observed to be concentrated around these vortical hotspots. The interface sandwiched by these structures undergoes further shear instabilities leading to a sharp rise in scalar mixing, that continues until the advent of the expansion wave.

**Figure 9:**
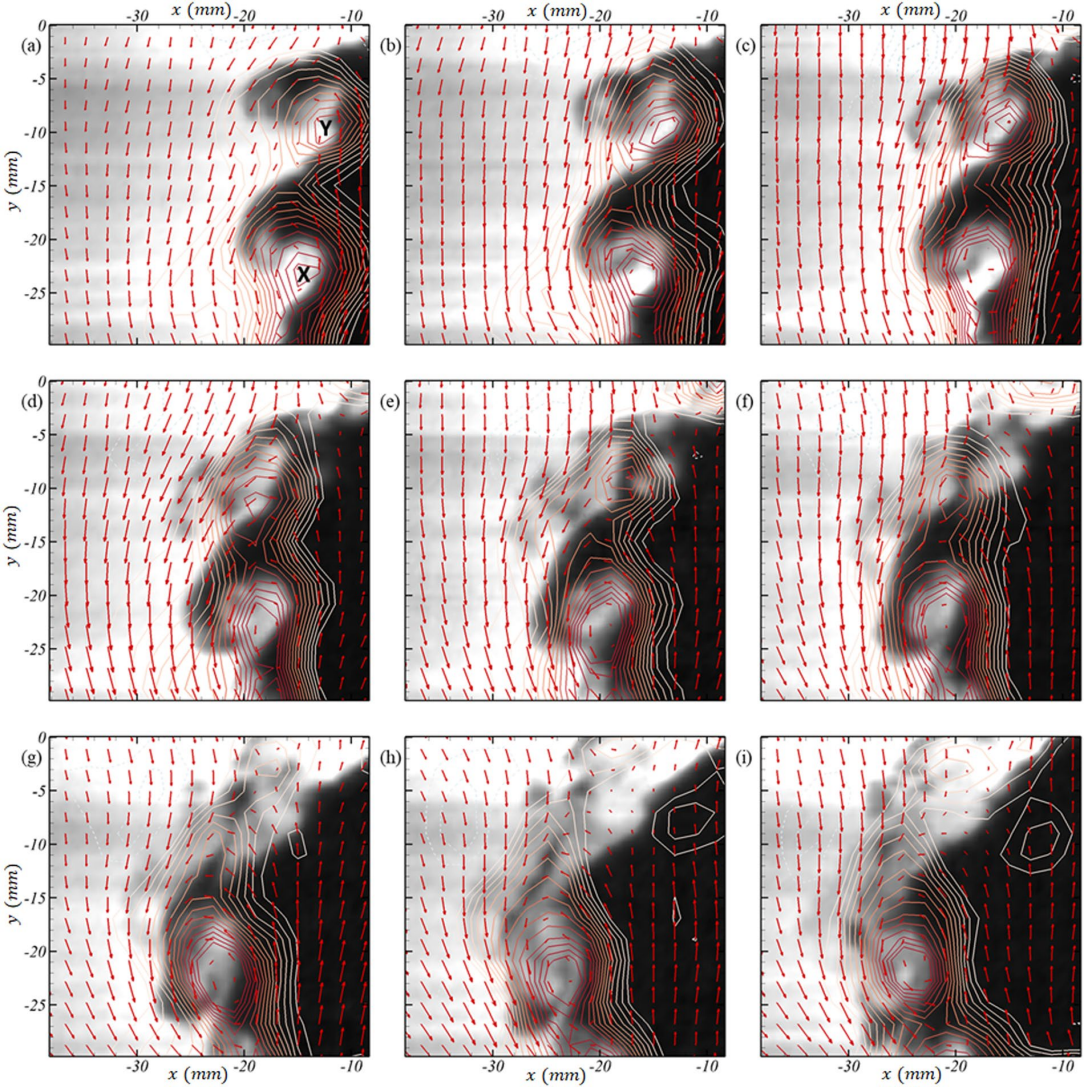
An illustrative temporal evolution of merging between two vortices [X and Y in (**a**)], and the associated scalar evolution shown in snapshots 67 *μs* apart, starting at 5.550 *ms* after incident shock in (**a**), 5.617 *ms* in (**b**), 5.684 *ms* in (**c**), 5.751 *ms* in (**d**), 5.818 *ms* in (**e**), 5.885 *ms* in (**f**), 5.952 *ms* in (**g**), 6.019 *ms* in (**h**) and 5.086 *ms* in (**i**). The vectors are velocities in the frame of reference of vortex-X (same scale as Fig. 4, in *m*/*s*) in (**a**) and line-contours represent vorticity with same levels as Fig. 8 (in *s*^−1^). See Supplementary video S4 for the evolution at full temporal resolution.

Molecular mixing in turbulent environments is an inherently small scale phenomena, and the utility of current measurements lies in the ability to track temporal evolution of these scales. Figure 9 shows an illustrative temporal sequence highlighting these small scale dynamics (see Supplemental video S4 for evolution at full temporal-resolution). The vorticity contours overlaid on the PLIF images identifies two vortices (X and Y marked in Fig. 9a). The velocities are marked in the frame of reference of the vortex X, showing the swirling motion around the same^46^. Both vortices are formed from the reshock-initiated vortex sheet, and the subsequent roll-up due to its unstable nature. Under their mutual influence, the symmetric-vortices approach each other and eventually merge^47^. This ‘mode-merging’ of vortices results not only in backward scatter of turbulence energy to smaller wave-numbers (large scales), but also in a local spike in scalar mixing as can be seen between Fig. 9f–i. This is the primary mixing mechanism in early stages of these vortex-dominated flows, and the mode merging has been studied theoretically (^48,48–50^, for eg.), experimentally (^48,49,51^, for eg.) and using computational tools^52^, for eg. However, existing studies on the effect of the mode-merging on scalar mixing (and specifically in variable density flows) are limited in scope and physics by the limitations of mixing models, or to low Reynolds numbers owing to the complexity of the same. The current high spatio-temporal resolution experiments are the first of its kind to capture this mode-merging and the induced mixing, specifically for shock-driven variable density phenomena. These advantages in the current diagnostics enable such studies related to temporal evolution of small scales that were previously not possible with conventional low speed approaches. Other possible directions currently being studied are the studies of vortex-accelerated vorticity deposition (VAVD^52^), vorticity-based mixing models, etc.

The current work demonstrates of the vortex dominated behavior of the turbulent mixing via time-resolved measurements of the vortex evolution. Further, the constraints on spatial resolution at these high speeds, especially in the velocity fields, is seen at instances where the PLIF images show vortical structures that are not captured individually by the vorticity fields owing to the lower resolution of the latter (see Supplementary video S3 at early times after reshock). This emphasizes the need for a coupled investigation using both high-resolution (low speed) and high-speed data, until such advances in imaging are available.

## Conclusion

The current work demonstrates the recent ability for spatio-temporally resolved measurements for shock-driven variable-density mixing, by exploiting the advances in diagnostics for simultaneous velocity and mixture fraction measurements. These enable future studies of turbulent mixing phenomena and modeling for applications in shock-driven hydrodynamics (such as shock- and blast-driven RMI and RTI in HED applications). Important experimental considerations are emphasized for these high-speed measurements, particularly related to the acquisition challenges, laser energy and flow characteristics. The quality of results was assessed and validated by comparing the results to previously published high-resolution, single-shot measurements on Richtmyer-Meshkov Instability. The high-speed vorticity fields enable tracking and temporal analysis of small scale vortical features that are essential to understand the molecular mixing behavior of such turbulent flows. These simultaneous diagnostics are essential to these flows, which are inherently dominated by small scale physics, as the same cannot be achieved using time-uncorrelated independent experiments (owing to randomness in small-scale structures) or simulations (owing to high spatio-temporal requirements and modeling constraints). Two commonly used models for initiation of deposited vorticity (SRZ98^42^ and WCB13^38^) for computational studies were evaluated with this unique dataset, and it was shown that the WCB13 approach is capable of offsetting the typical assumptions (to a limited extent) via a prescribed interface jump velocity when the same is available via measurements. Further, an illustration of the often-studied symmetric vortex merging^47^ was demonstrated in a shock-driven variable-density flow for the first time, together with the induced effects on scalar mixing in an unprecedented detail. Detailed analysis on non-stationary physics such as the Vortex Accelerated Vorticity Deposition (VAVD^52^), vorticity-based stochastic modeling of mixing, etc. are made possible with these techniques, that were previously not amenable to experimental methods. These measurements are one of the first of this kind for high-speed turbulent mixing applications, and highlight the exciting possibilities that the recent advances in diagnostics enable.

## Supplementary information

supplementary video 1.

supplementary video 2

supplementary video 3

supplementary video 4
